# Modulation of Auditory Cortex Response to Pitch Variation Following Training with Microtonal Melodies

**DOI:** 10.3389/fpsyg.2012.00544

**Published:** 2012-12-05

**Authors:** Robert J. Zatorre, Karine Delhommeau, Jean Mary Zarate

**Affiliations:** ^1^Montreal Neurological Institute, McGill UniversityMontreal, QC, Canada; ^2^International Laboratory for Brain, Music, and Sound ResearchMontreal, QC, Canada; ^3^Department of Psychology, New York UniversityNew York, NY, USA

**Keywords:** auditory cortex, perceptual learning, pitch perception, functional MRI, individual differences

## Abstract

We tested changes in cortical functional response to auditory patterns in a configural learning paradigm. We trained 10 human listeners to discriminate micromelodies (consisting of smaller pitch intervals than normally used in Western music) and measured covariation in blood oxygenation signal to increasing pitch interval size in order to dissociate global changes in activity from those specifically associated with the stimulus feature that was trained. A psychophysical staircase procedure with feedback was used for training over a 2-week period. Behavioral tests of discrimination ability performed before and after training showed significant learning on the trained stimuli, and generalization to other frequencies and tasks; no learning occurred in an untrained control group. Before training the functional MRI data showed the expected systematic increase in activity in auditory cortices as a function of increasing micromelody pitch interval size. This function became shallower after training, with the maximal change observed in the right posterior auditory cortex. Global decreases in activity in auditory regions, along with global increases in frontal cortices also occurred after training. Individual variation in learning rate was related to the hemodynamic slope to pitch interval size, such that those who had a higher sensitivity to pitch interval variation prior to learning achieved the fastest learning. We conclude that configural auditory learning entails modulation in the response of auditory cortex to the trained stimulus feature. Reduction in blood oxygenation response to increasing pitch interval size suggests that fewer computational resources, and hence lower neural recruitment, is associated with learning, in accord with models of auditory cortex function, and with data from other modalities.

## Introduction

Learning takes many forms and hence manifests itself in a variety of ways throughout the nervous system. An intensely studied form of learning involves the adjustments that occur in perception as expertise develops with a given class of stimuli or in a given domain. There is a long history of behavioral research showing that training enhances the ability to perceive small differences in stimulus features, usually termed perceptual learning; for review see Wright and Zhang (2009). At the neural level, learning-induced improvements in perceptual thresholds are often attributed to changes in cortical organization, such that experience with a specific stimulus set leads to an enhanced or expanded representation in the corresponding portion of sensory cortex; this pattern is often though not always: see Brown et al. (2004) reported in neurophysiological studies of auditory learning in animals (Recanzone et al., 1993; Polley et al., 2006); for reviews see Buonomano and Merzenich (1998), Irvine (2007).

Many studies of human auditory learning report that training leads to greater amplitude of certain evoked potential components from auditory cortex (AC; Kraus et al., 1995; Bosnyak et al., 2004; Lappe et al., 2008) even after brief training (Alain et al., 2007), or to greater hemodynamic signal from AC and/or associative areas (Golestani and Zatorre, 2004; Dehaene-Lambertz et al., 2005; Gaab et al., 2006). However, the model of cortical expansion may not necessarily apply in a straightforward way to all perceptual learning (Kilgard et al., 2001). Indeed, the neuroimaging literature on changes in cortical processing with learning shows many different patterns, including both increases and decreases of activity in sensory, motor, and association cortical regions (Kelly and Garavan, 2005; Ohl and Scheich, 2005; Steele and Penhune, 2010). This heterogeneity is also evident in cross-sectional studies of musical training (Pantev et al., 1998, 2001; Schneider et al., 2002; Koelsch et al., 2005; Zarate and Zatorre, 2008; Margulis et al., 2009).

Melodies provide a particularly rich paradigm for studying higher-order auditory perceptual learning. Behavioral evidence indicates that melodies are encoded in terms of intervallic relationships between pitches (Attneave and Olson, 1971), and hence rely on configural processing (Divenyi and Hirsh, 1978; Dowling, 1978). Our aim in the present study was to develop a training procedure that would emphasize configural learning, by requiring musically untrained listeners to distinguish one melody, or pattern of tones, from another; we accomplished this by using microtonal melodies (“micromelodies”), i.e., melodies in which the intervals are much smaller than those used in conventional Western music (Parncutt and Cohen, 1995). There are early reports that after sufficient exposure, listeners report perceiving melodic interval relationships with intervals on the order of 90% smaller than usual (Werner, 1940), but no formal learning data have previously been reported, and no imaging studies of melody perceptual learning exist.

Another reason for selecting melodies as a means to probe perceptual learning is that neuroimaging research has clarified the functional substrates of melody perception, which seems to rely on a hierarchical network of auditory cortices, involving both anterior and posterior portions of the superior temporal gyrus (STG), with a right-sided predominance (Zatorre, 1985; Griffiths et al., 1998; Patterson et al., 2002). But because melodic processing also typically involves working memory and other non-specific mechanisms, extratemporal regions are also frequently involved, particularly in the dorsolateral frontal and parietal cortices (Zatorre et al., 1994; Griffiths et al., 1999; Gaab et al., 2003; Brown and Martinez, 2007; Foster and Zatorre, 2010b). In a study especially relevant here, Hyde et al. (2008) showed that as the size of pitch changes increased in a simple melodic pattern, there was a concomitant increase in neural activity as measured via functional magnetic resonance imaging (fMRI) in a portion of the right planum temporale adjacent to lateral Heschl’s gyrus. This pattern likely arises because if there is sensitivity of neural activity to a given feature, in this case pitch change, there is increased neural recruitment as that feature becomes more salient. Here, we took advantage of this paradigm and expected that this region would be maximally sensitive to manipulations of pitch intervals in micromelodies, and perhaps also to learning thereof.

Based on the foregoing, we predicted that listeners should be able to improve their perception of micromelodies after sufficient training; importantly, based on earlier behavioral studies (Demany, 1985; Delhommeau et al., 2002, 2005; Ari-Even Roth et al., 2003), we expected that complete generalization would occur for frequencies other than those trained, demonstrating that learning is not confined to one frequency region, but rather involves higher-order, configural processing. We also tested generalization to a new task, involving melody transposition, that was not explicitly trained. These predictions were examined by recruiting two groups of individuals, one of whom received micromelody training over a 2-week period, and a control group which did not; both groups were tested using discrimination tasks before and after the training period. The trained group also underwent fMRI scanning before and after the training period. We predicted that fMRI would reveal training-induced modulation in right auditory cortical areas previously associated with melodic processing, and perhaps in frontal or parietal areas involved in other aspects of the task as well. Importantly, the experimental design allowed us to distinguish global changes related to learning the task from changes in the auditory cortical responses directly linked to the size of the pitch interval, since the latter were systematically varied in a parametric fashion, as in Hyde et al. (2008), allowing us to search for a learning-induced change specifically in that response.

## Materials and Methods

### Subjects

Twenty healthy volunteers (12 female) were recruited from the McGill University community. All subjects (mean age = 22 ±  4.4 years old) were right-handed, had normal hearing, and were devoid of neurological or psychological disorders and contraindications for fMRI. All subjects gave informed consent to participate in this study, in accordance with procedures approved by the Research Ethics Committee of the Montréal Neurological Institute. All subjects were classified as non-musicians because they had less than 3 years of vocal and/or musical training or experience, and were not currently practicing or performing music. The subjects were randomly divided into two groups of 10 people each: an experimental group that received auditory training and scanning, and a control group that was neither trained nor scanned.

### Global procedure

**Figure 1:**
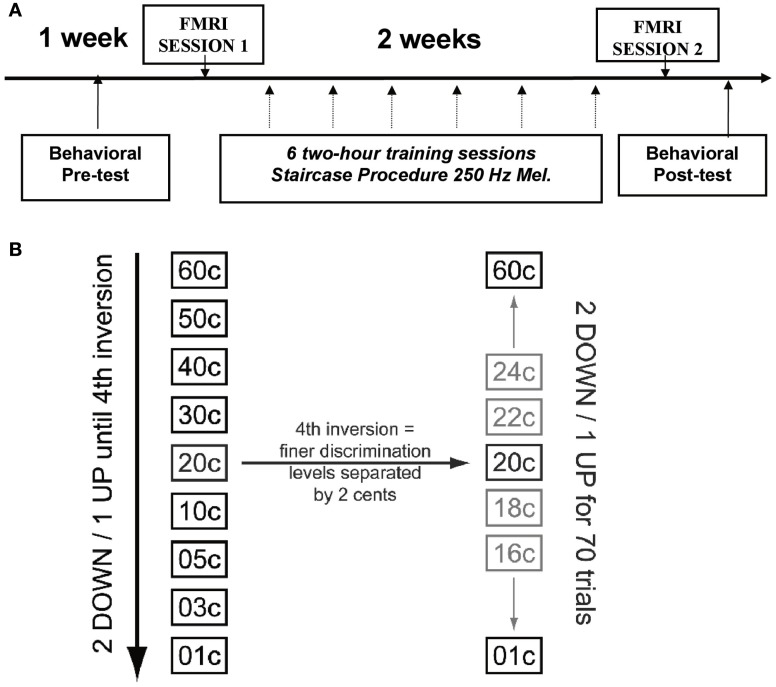
**(A)** Time line of experimental procedure showing relative timing of pre- and post-training behavioral tasks and learning procedures, as well as fMRI scanning. **(B)** Illustration of staircase procedure. The stimuli were chosen from the left column (coarser spacing of pitch intervals) during the first part of the procedure, until the fourth reversal was achieved; subsequently the stimuli were chosen from the more finely spaced stimuli (2 cents difference) indicated in the right column.

Figure 1A depicts the general timeline for all testing and training sessions. The experimental group was tested with a battery of auditory discrimination tasks to determine baseline performance levels. These subjects also performed a subset of these same auditory discrimination tasks in the scanner to obtain neuroimaging data. After the first scanning session, the experimental group was given auditory discrimination training across six sessions spread over 2 weeks using an adaptive paradigm (see Auditory Discrimination Training and Tasks). Following training, these subjects once again performed the same subset of auditory discrimination tasks in the scanner as in the first scan. Finally, the trained subjects were tested behaviorally one last time after scanning, with the larger set of auditory discrimination battery used prior to training. Control subjects were tested behaviorally with the same battery of auditory tasks twice, in the same task order as the trained subjects, with approximately 16 days between the sessions to match the amount of time between initial and final behavioral testing in the trained subjects; they were not scanned. The same two groups of individuals were also tested on vocal production tasks in a separate study (Zarate et al., 2010); the control sample was tested behaviorally at two time points, while the trained group was tested both behaviorally and with fMRI performing a vocal task. All of the vocal fMRI tasks were performed after the fMRI micromelody perception tasks from the present study had been completed, however, so that those tasks cannot have influenced the present data.

### Equipment

For the behavioral testing sessions, each subject sat in front of a computer screen and wore a pair of headphones (Sony MDR-V900, New York, NY, USA), through which all auditory stimuli were delivered binaurally at a comfortable intensity. Stimuli were presented via a personal computer using Presentation software (Neurobehavioral Systems, Inc., Albany, CA, USA). During scanning sessions, subjects in the experimental group were tested in a Siemens Sonata 1.5-T magnetic resonance (MR) scanner. Stimuli were delivered via MR-compatible headphones (Commander XG headset, Resonance Technology, Inc., Northridge, CA, USA). All visual cues were back-projected onto a screen at the subjects’ feet, viewed via a mirror attached to the head coil.

### Stimuli

**Figure 2:**
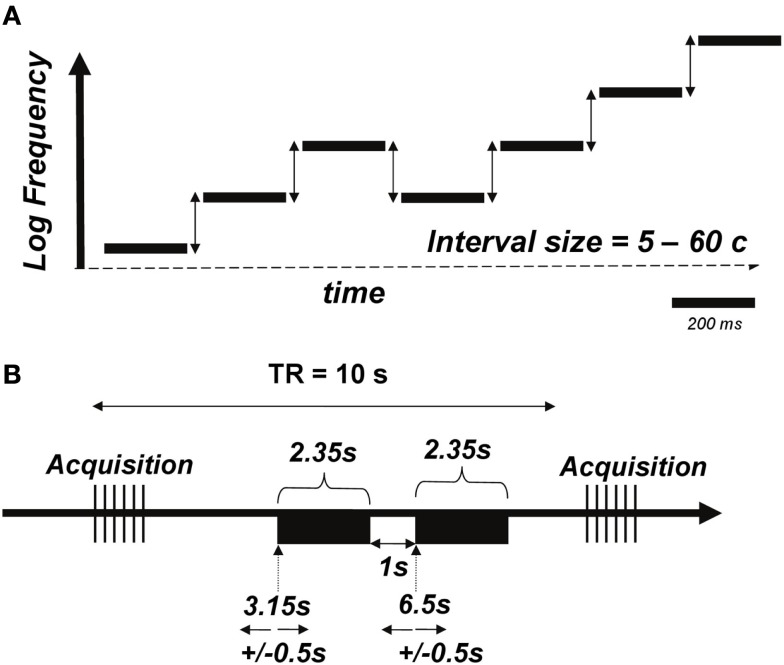
**(A)** Schematic of the micromelody stimuli. Each of seven tones was generated such that there would be two or three inversions of melodic contour; the middle tone was set at 250 Hz for the training stimuli (or 500 or 1150 Hz for pre- and post-training generalization testing). The intervals between notes (vertical arrows) was varied parametrically between 5 and 60 cents. Sound files (attached) provide examples of the stimuli; the first file corresponds to a micromelody with 5 cents intervals, the second with 20 cents intervals, and the third with 60 cents intervals. **(B)** Schematic of the functional MRI acquisition protocol. A pair of micromelodies (denoted by black rectangles) was presented in the quiet interval between volume acquisitions using a 10-s TR. Onsets of the first and second melodies occurred at 3.15 and 6.5 s, respectively, with an additional jitter of 0.5 s imposed.

We used micromelodies as the main stimuli for auditory discrimination training and testing (Figure 2A; sound files with examples of stimuli can be found as supplementary material). We define micromelodies as melodies with pitch intervals (frequency ratios) that are smaller than 100 cents (the cent scale is used to represent logarithmic frequency differences; 100 cents corresponds in musical terminology to a semitone, the smallest interval in the Western musical scale). Thus, each micromelody was made up of intervals substantially smaller than those that listeners would normally have been exposed to in ordinary music. Micromelodies consisted of seven sinusoidal tones, each of which was 200 ms long, with an inter-tone interval of 150 ms, and 50 ms of silence at the end of the melody; total length of each micromelody was therefore 2.35 s. There was an inter-stimulus interval of 1 s within each pair of micromelodies presented for discrimination. The middle tone (i.e., number 4 of 7) of each micromelody was set to the training frequency of 250 Hz or to non-trained frequencies of 500 or 1150 Hz to test for generalization. Micromelodies were constructed with either zero or at most one consecutive repetition of any given note, and, to create enough variety, with either two or three inversions of melodic contour (e.g., down-down-down-**up**-**down**-down-**up** would contain three inversions, denoted in boldface).

### Auditory discrimination training and tasks

The auditory discrimination task was a two-alternative, forced-choice procedure in which subjects were presented with two micromelodies in succession, and were required to indicate whether they were the same or different; during discrimination testing, subjects did not receive feedback on whether or not their answer was correct. We used seven different interval scales for behavioral testing outside the scanner, such that the intervals between successive tones in each micromelody were either: 5, 10, 15, 20, 30, 40, or 60 cents; micromelodies at each interval scale were presented randomly during testing. Any given micromelody only used one consistent value from amongst these seven intervals, such that all intervals within a melody were the same size. On half of the discrimination trials, both micromelodies within a presented pair were the same; in the other half the stimuli differed. On these different trials the micromelodies were matched for interval scale (e.g., both consisted of 20 cents intervals, or both of 60 cents intervals), but the second item was randomly selected from the pool of items such that it had a different melodic contour than the first item (thus, more than one note differed between the two micromelodies).

Subjects were asked to perform a same/different discrimination under five different conditions using a conventional method of constant stimuli. The first condition presented micromelodies all centered at 250 Hz from the pool that was eventually used for discrimination training. A second condition used a different subset of 250-Hz micromelodies that were not used during training sessions. The third and fourth conditions presented micromelodies at the two non-trained frequencies of 500 and 1150 Hz, respectively. The final condition presented in each pair a trained 250-Hz micromelody and another micromelody centered at the 1150-Hz frequency, and subjects were asked to determine whether the micromelody contour was the same or different after the frequency transposition (referred to as the “transposed” task).

After the first behavioral and fMRI testing sessions, the experimental group only went through six training sessions of micromelody discrimination at 250 Hz on separate days, spread evenly across 2 weeks. In contrast to pre- and post-training discrimination testing sessions, during the training sessions subjects performed an adaptive procedure and received visual feedback for their answers on every trial. Training sessions used a two-alternative, forced-choice staircase procedure with a “2 down-1 up” adaptive level variation rule (Figure 1B). After two successive trials that were correctly answered, the difficulty level would increase (e.g., go down from 60 to 50 cents), and for each trial that was answered incorrectly, the difficulty level would decrease (e.g., go up from 50 to 60 cents). This adaptive procedure would continue until four reversals in difficulty occurred, resulting in a variable number of trials per subject in each session. Subsequently, finer discrimination training took place over 70 trials, starting at the interval size that evoked the fourth reversal; each difficulty level was separated by only 2 cents during this portion of training (Figure 2B). This procedure allowed us to establish a threshold for performance for each run. Each of these runs was repeated 10 times during each training session, and the outcome of the 10 runs was averaged to represent the threshold achieved on that day.

Following training, subjects in the experimental group were tested again for discrimination using the identical procedure to that used before training, as described above (i.e., method of constant stimuli, without feedback), in order to determine the effect of training, and to examine whether training at 250 Hz would also generalize to the micromelodies centered at non-trained frequencies. Subjects in the (untrained) control group received the same set of discrimination tests with a similar intervening time period.

### fMRI protocol

Prior to functional scanning, a high-resolution (voxel size = 1 mm^3^) T1-weighted scan was obtained for anatomical localization. During the two functional runs, whole-head frames of 25 contiguous T2*-weighted images aligned with the Sylvian fissure were acquired in an interleaved fashion (TE = 85 ms, TR = 10 s, 64 × 64 matrix, voxel size = 5 × 5 × 5 mm^3^, FOV = 320 mm^2^) on a 1.5-T Siemens scanner. We utilized a sparse-sampling experimental design, in which tasks were performed during the silences between image acquisitions to prevent scanner noise from interfering with the auditory stimuli (Belin et al., 1999). On each trial, the micromelodies were presented at 3.15 and 6.15 s following the previous scan acquisition, and these presentation times were systematically jittered by ±500 ms to maximize the likelihood of obtaining the peak of the hemodynamic response for each task (Figure 2B). Within each run, listeners were presented with three blocks of conditions: (1) a block of passive auditory stimulation, during which a pair of identical micromelodies (at 5, 10, 15, 20, 30, 40, and 60 cents), as well as monotonic sequences (i.e., 0 cents), were presented; (2) a block of active micromelody discrimination using only 5, 15, and 30 cents interval scales; and (3) another block of passive auditory stimulation. The active task used only three small-interval values because we knew that behavioral performance would be close to ceiling for items above 30 cents, and we wanted to test within a range sensitive to learning. The pertinent interval scales were pseudorandomized within each passive and active block. Six trials of silence, during which baseline activity could be measured, were also presented in a pseudorandom manner during each of the three blocks. Therefore, a total of 128 trials of passive auditory stimulation, 60 trials of active discrimination, and 36 trials of silence were presented. The passive and active runs had identical presentation parameters, with the difference being that the melodies were different on half the trials in the active task, whereas they were always identical during the passive task; subjects were informed of which block of trials was coming up prior to each condition via a visual cue.

### Behavioral analyses

For control and experimental groups, micromelody discrimination performance for each condition was assessed at each time point (pre- and post-training) by determining the percentage of trials that each subject answered correctly. The percentages were analyzed using four-way repeated-measures analyses of variance (ANOVAs), with group as the between-subjects variable, and time (pre- versus post-training), condition (250 Hz-trained, 250 Hz-non-trained, 500, 1150 Hz, or transposed), and micromelody interval scale (5, 10 cents, etc.) as within-subject variables. A more focused three-way repeated-measures ANOVA (group by time by condition) was performed with discrimination scores collapsed across all interval scales, as well as an ANOVA analyzing the effects of group, time, and interval scale only at the condition with the trained 250-Hz micromelodies. We also performed a two-way repeated-measures ANOVA on the adaptive behavioral data from the training sessions with the experimental group, using session (six in total) and run (10 within each session) as within-subject variables. Simple effects tests were used to analyze significant interactions, and the Bonferroni test was used for all *post hoc* comparisons.

### fMRI analyses

To correct for motion artifacts, all blood-oxygen-level-dependent (BOLD) images were realigned with the third frame of the first functional run using the AFNI software (Binder et al., 1996). To increase the signal-to-noise ratio of the imaging data, the images were spatially smoothed with a 12-mm full-width at half-maximum isotropic Gaussian kernel. For each subject, we conducted our image analyses in a similar fashion to that described in a previous paper (Zarate and Zatorre, 2008), using fMRISTAT, which involves a set of four Matlab functions that utilize the general linear model for analyses (Worsley et al., 2002). Before group statistical maps for each contrast of interest were generated, in-house software was used to linearly transform anatomical and functional images from each subject into standardized MNI305 stereotaxic coordinate space (Talairach and Tournoux, 1988; Collins et al., 1994). We performed covariation analyses independently within each test session (pre- and post-training) and with each task (passive listening and active discrimination); in these analyses, the input variable was the size of the micromelody interval scale (passive: 0–60 cents; active, 5, 15, or 30 cents), which was regressed on the imaging data on a voxel-by-voxel basis to find brain regions in which BOLD signal changed as a linear function of interval size.

To determine learning effects (i.e., post-training versus pre-training), we first statistically compared the post-training data with the pre-training data using a fixed-effects linear model in each subject. We subsequently combined these results across all subjects with a mixed-effects linear model. Significant peaks of activity are reported when they exceed a whole-brain false-discovery rate (FDR) of *p* < 0.05 (Genovese et al., 2002) as calculated within each contrast. For regions of AC active in the pre-training data, we also report any changes between sessions that exceed the FDR threshold based on a mask of these active areas in the pre-training session; other findings are reported as warranted. For descriptive purposes, voxel-of-interest (VOI) analyses were performed on voxels that displayed peak activity in group-contrasted BOLD images. For each voxel in MNI305 space, the BOLD signal is extracted from the same voxel in standardized space within each subject. At each VOI, the BOLD signal for the task of interest is calculated as a percentage of change of BOLD signal during the baseline condition in the following way: (BOLD signal during task – BOLD signal during baseline)/(BOLD signal during baseline) × 100. These values were also used to generate slopes for each individual in order to test hemispheric differences.

## Results

### Behavioral results

**Figure 3:**
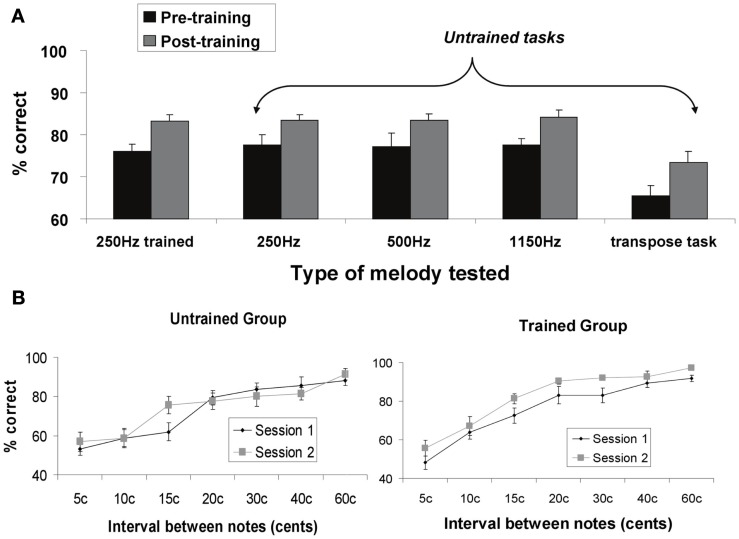
**(A)** Percent correct behavioral performance on micromelody discrimination tasks pre- and post-training for the trained group. The first set of bars represents performance before (Session 1) and after training (Session 2) using the same materials as used in the training (micromelodies centered at 250 Hz). Subsequent bars represent stimuli and tasks that were different from those used during training: micromelodies centered at 250 Hz (but not the same items as used in training); micromelodies centered at 500 and at 1150 Hz; and a discrimination task in which the second item was transposed to a different pitch level (i.e., 1150 Hz). There was significant and comparable improvement in all tasks after training, indicating generalization to new pitch levels and new tasks. **(B)** Performance on the 250 Hz micromelodies before and after training as a function of pitch interval for both the control (untrained) group, and the experimental (trained) group. No change was observed at any pitch level for the control group. Significant improvement was obtained in the trained group across all intervals.

We measured percent correct scores for the two groups (experimental and control) at each time point (before and after training), for the five micromelody discrimination conditions at each of the seven interval scales. The behavioral data for the five different discrimination tasks are shown in Figure 3A; the data for the 250 Hz micromelody task broken down according to interval scale are shown in Figure 3B. The overall pattern revealed the expected learning in the trained group but not the control group. Learning generalized across all tasks.

The four-way mixed ANOVA described above revealed significant main effects of all four factors (all *p*s < 0.05), as well as significant two-way interactions between group and time [*F*(1, 18) = 8.25, *p* = 0.01], time and interval scale [*F*(6, 108) = 2.64, *p* < 0.05], and condition and interval scale [*F*(24, 432) = 2.17, *p* < 0.01]; no other interactions were statistically significant (all *p*s > 0.1). As expected, there was a large main effect of interval scale, which merely reflects the fact that performance improved systematically as the size of the intervals increased (Figure 3B). The main effect of condition is attributable to the melody transposition task, which was more difficult than any of the others.

The most relevant effect from this analysis is provided by the group by time interaction, since it tests for the specific effect of learning. Simple effects tests performed on this interaction determined that discrimination training significantly enhanced micromelody discrimination in the experimental group compared to their baseline performance (*p* < 0.001), and that this effect generalized across all tasks (Figure 3A), whereas the controls showed no significant change at the end of the experiment compared to the first testing session. Similarly, whereas there was no significant difference in micromelody discrimination between the control and experimental groups at the beginning of the experiment, the experimental group performed significantly better than the control group across all conditions and interval scales (Figure 3B) after training (*p* < 0.01). It is of interest to note that although generalization was seen here to all perceptual tasks, we did not observe any evidence of training-induced enhancement in vocal production tasks in the same individuals (Zarate et al., 2010).

**Figure 4:**
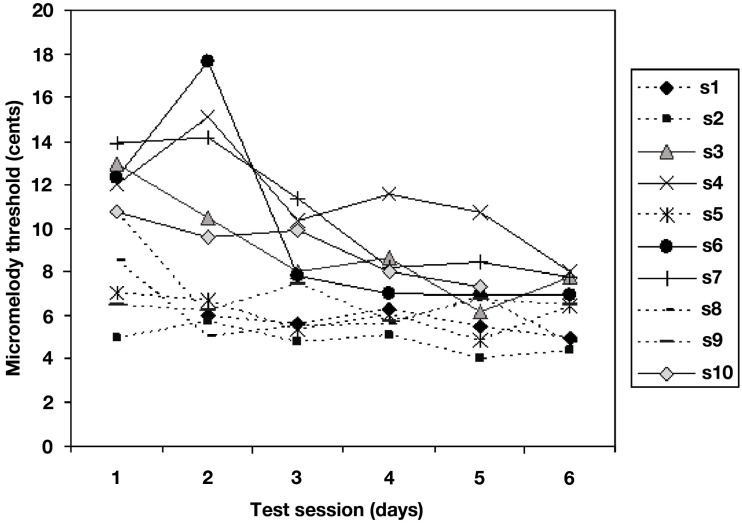
**Mean micromelody discrimination thresholds (in cents) obtained during adaptive staircase procedure with feedback across 6 days of learning**. Each line represents data for one individual. Five of the ten achieved asymptotic performance on the first or second day (termed faster learners, dotted lines); the other five (slower learners, solid lines) showed more gradual improvement over the 6-day period. The two subgroups did not differ in terms of final performance.

The two-way ANOVA performed on the adaptive behavioral data obtained during training (Figure 4) demonstrated, as expected, a large effect of training session [*F*(5, 45) = 5.69, *p* = 0.0004], attributable to the fact that thresholds dropped very significantly across sessions. However, closer inspection of the individual data revealed considerable heterogeneity in the learning curves. We addressed this issue in two ways. First, we divided the group into two: whereas some individuals (5 of the 10) showed a gradual decline in thresholds from the first to the sixth days (hereafter termed slower learners), the other five showed little or no change across sessions, due to the fact that those individuals were already close to an asymptotic maximal performance (thresholds around 5 cents on the first or at most second day of training), suggesting that their learning was so rapid that it was already complete as of the first day or two, and there was no further improvement after that (this subgroup is hereafter termed faster learners). Second, we quantified this variability in a direct, unbiased fashion by computing the linear slopes of the functions relating threshold values on each run of learning across days for each individual.

Importantly, the two subgroups did not differ significantly in performance on the micromelody discrimination task either at pre- or at post-training; nor was the correlation between learning slope and performance on pre-training significant (*r* = 0.30, *p* > 0.10). This indicates that the differences between slower and faster learners emerged only after the pre-training testing period (that is, during early learning), and that whatever differences existed in the speed of learning, final performance was equivalent across subgroups. Conversely, the correlation between each individual’s learning slope and mean threshold value on day 1 of learning was highly significant (*r* = −0.91, *p* < 0.001), which indicates that those who showed a steep learning function (slower learners) started off with a high threshold, whereas those with a flatter learning curve (faster learners) were already close to ceiling performance on the first day of training, as may be seen in Figure 4.

### Neuroimaging results

Our principal interest was to determine how training modifies the pattern of brain activity specifically associated with the processing of pitch intervals, as opposed to the global neural response to sound, or to mere familiarity with the stimuli. Prior to determining effects of training, therefore, the first step in the analysis was to measure the effect of the variation in size of pitch interval scales on auditory cortical responses via covariation analyses between the input variable of pitch interval size and BOLD signal within each task (passive listening and active discrimination) in each test session (pre- and post-training) independently.

#### Pre-training pitch covariation

**Figure 5:**
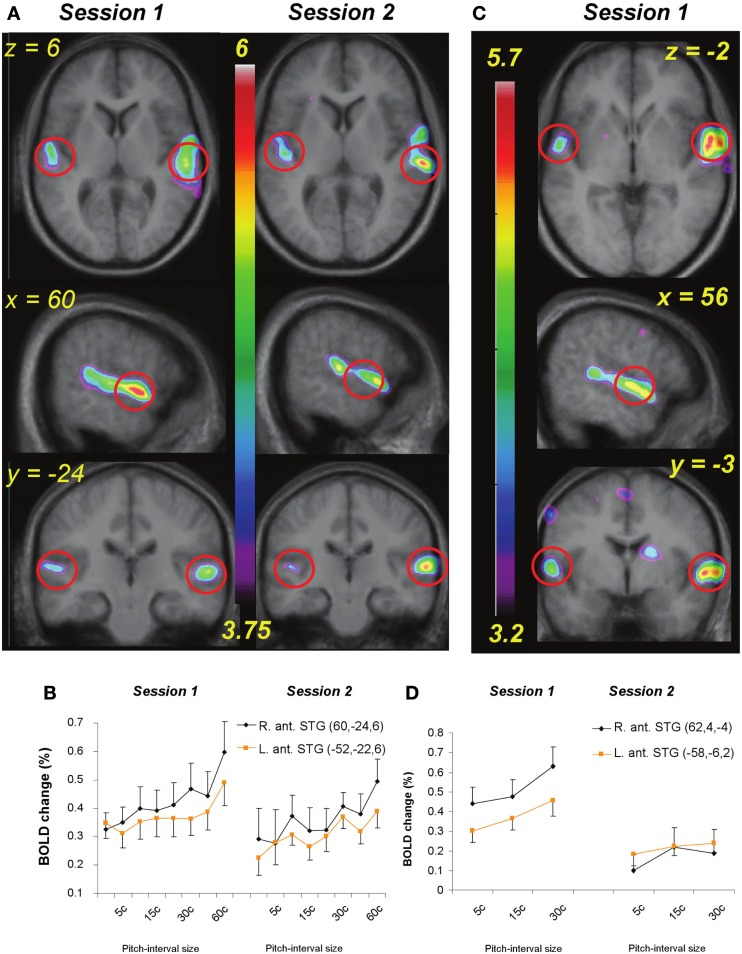
**(A)** functional MRI data for the passive listening task before and after training (Session 1 and 2) shown in horizontal, sagittal, and coronal slices (top to bottom). Statistical parametric maps (color scale refers to *t*-values) indicate strength of the covariation between BOLD signal change and increasing pitch interval size of micromelodies; changes were confined to the superior temporal gyrus (STG). **(B)** Extracted BOLD values for two regions located in right and left anterior STG (indicated approximately by red circles in **(A)** to illustrate change in BOLD as a function of interval size in the passive condition. The slope of the function is steeper on the right; after training there is a slight drop in the slope. **(C)** As in **(A)** but for the active task, pre-training only (as no significant changes were detected after training). **(D)** As in **(B)**, showing BOLD signal as a function of the interval sizes used during the active task in two anterior STG areas; positive slopes found pre-training reduced to no significant slope after training.

Looking first at the pre-training passive task results, the analysis revealed widespread covariation responses throughout much of the STG of both hemispheres, including portions of Heschl’s gyrus, the planum temporale, and the planum polare (Table 1; Figure 5A, Session 1). Thus, these regions exhibited increasing BOLD activity as a function of increasing pitch interval size. No extratemporal covariation of BOLD signal was observed with these analyses, but there was significant change in the negative direction (i.e., less BOLD activity as pitch intervals increase) in a left superior frontal region; a similar negative relationship was observed in midline visual cortex (Table 1). The latter effect is most likely a consequence of cross-modal interactions between increased auditory cortical activity resulting in decreased visual cortical activity (Johnson and Zatorre, 2005).

**Table 1:** **Pre and post-training covariation analysis. Passive condition**.

Region	Pre-training Coordinates			*t*-Value	Post-training Coordinates			*t*-Value
	*x*	*y*	*z*		*x*	*y*	*z*	
**BOLD SIGNAL INCREASES**								
Right anterior STG	58	−2	−2	5.93	66	0	2	5.14
					62	6	−2	5.12
					50	0	−6	5.12
Right planum temporale	60	−24	6	5.18	66	−26	8	5.53
	58	−20	6	5.15				
Right posterior STG	62	−38	14	5.04	–	–	–	
Left anterior STG	−56	−10	4	4.99	−50	−8	2	4.60
Left planum temporale/Heschl’s gyrus	−58	−30	12	5.10	−48	−18	6	4.61
	−52	−22	6	4.59				
**BOLD SIGNAL DECREASES**								
Visual cortex	−2	−94	18	−4.76	12	−64	12	−4.00
	8	−80	18	−4.10	−4	−52	20	−3.73
	−20	−96	18	−3.50	12	−56	22	−3.64
					−18	−100	12	−3.48
					4	−56	22	−3.47
Right superior frontal gyrus	26	52	36	−3.57	8	54	40	−3.59
					14	56	42	−3.48
Left superior frontal gyrus	−26	48	42	−3.34	−12	48	30	−5.05
					−20	58	34	−4.20
					−20	56	28	−4.17
					−28	12	48	−3.85
					−38	12	58	−3.38
Left frontal pole	−20	54	4	−3.56	–	–	–	
	−20	60	12	−3.44				
Right hippocampus	26	−2	−32	−3.42	–	–	–	

As predicted, the covariation effect within the STG appeared more extensive in the right than on the left, and also of higher magnitude. To evaluate this possible asymmetry statistically, we selected roughly symmetrical peak positions in each STG from the pre-training data (MNI coordinates: 60, −24, 6 on the right, and either −52, −22, 6 or −58, −30, 12 on the left), extracted the BOLD signal values from each of these (relative to silence), and calculated the slope of the relation between BOLD and pitch interval for each individual for each peak. These slope estimates were then compared between the one right STG and the two left STG peaks using a paired-sample *t*-test; this analysis confirmed that the strength of the relationship was higher on the right than on the left [*t*(9) = 2.35, *p* = 0.02 for the first peak contrast; *t*(9) = 1.80, *p* = 0.05 for the second peak contrast] in the pre-training data set. Although the slope estimates themselves could be considered as biased due to non-independence of the region of interest selection, there is no particular bias in this respect between left- and right-sided regions; on the contrary, the maximal slopes on each side are compared via this procedure. A similar trend was present when this same analysis was carried for the post-training data (Figure 5A, Session 2), but it was not significant.

In the pre-training active condition, the overall pattern was similar to that observed in the passive condition, in that there was increased STG BOLD response as a function of increasing pitch interval size (Table 2; Figure 5B); but whereas the BOLD covariation response was quite extensive throughout the right STG from anterior to posterior locations, it was much more restricted on the left, with only a single peak in an anterior location reaching significance. Comparing the slopes of the responses within this left STG region (−58, −6, 2) with its closest homolog on the right STG (60, 4, 0) confirmed a right-sided advantage [*t*(9) = 1.97, *p* = 0.04]; this analysis however underestimates the size of the asymmetry since there were no left STG peaks to compare with the many spatially distributed right STG foci observed (Table 2). In addition to the STG modulation, we also observed responses in the basal ganglia, supplementary motor area, and premotor cortex. Negative responses were similar to those seen in the passive condition, including superior frontal gyrus, and posterior cortical areas.

**Table 2:** **Pre- and post-training covariation analysis. Active condition**.

Region	Pre-training Coordinates			*t*-Value	Post-training Coordinates			*t*-Value
	*x*	*y*	*z*		*x*	*y*	*z*	
**BOLD SIGNAL INCREASES**								
Right anterior STG	62	4	−4	5.69	62	−6	4	3.38
	60	−4	0	5.43				
Right planum temporale	62	−20	4	5.01	70	−18	2	3.78
	70	−20	4	4.83	64	−14	2	3.67
Right posterior STG	52	−38	8	4.73	–	–	–	
Left anterior STG	−58	−6	2	5.14	−58	−6	4	3.12
Right dorsolateral frontal	54	0	42	3.38	–	–	–	
Left superior frontal	−24	−12	64	3.59	−28	−10	58	4.21
					−48	−22	52	3.46
					−44	−30	58	3.42
SMA	0	−6	60	3.94	−6	−8	52	3.74
	−6	2	56	3.38
Left posterior cingulate	−48	−36	34	3.57	−54	−28	24	4.23
					−52	−32	32	3.90
Right caudate	18	0	16	4.29	–	–	–	
Left caudate	−16	4	18	3.37	–	–	–	
Left putamen	−20	4	0	3.43	–	–	–	
**BOLD SIGNAL DECREASES**								
Right superior frontal gyrus	40	54	16	−3.79	–	–	–	
Left superior frontal gyrus	−20	48	40	−3.61	–	–	–	
	−24	42	30	−3.35

#### Effects of training on pitch covariation

Having determined the neural responses specifically associated with processing of microtonal intervals places us in a position to evaluate the specific effects of training. Covariation analysis of the post-training data separately from the pre-training data showed that for the passive task, the BOLD patterns were similar to those obtained pre-training, but with slightly weaker modulation of BOLD in the STG bilaterally (Table 1; Figure 5A, Session 2); for the active task, there was considerably less BOLD covariation response overall, and consequently less of an asymmetry between left and right (Table 2; Figure 5B). None of the post-training effects in the active condition reached the FDR threshold of significance (but sub-threshold values are shown in Table 2 for comparison), indicating that there was little change in activity in relation to larger pitch intervals after training.

To determine the changes that occurred as a function of training directly in a principled manner, we computed a contrast between post- and pre-training of the covariation analyses. Positive changes in this analysis indicate brain regions whose activity increased more as a function of pitch interval size after than before training; negative values indicate that the response to pitch interval size was lower after than before training. The contrast for the passive condition did not yield any statistically significant changes; but there were two regions of note in the left and right planum temporale/STS region (−56, −46, 14; *t* = 3.18 and 54, −36, 4, *t* = 2.8) which showed lower covariation after training; although not meeting the FDR threshold, their bilateral placement, and their similarity to areas observed in the active condition, as discussed below, suggest they may not be false-positive responses.

**Figure 6:**
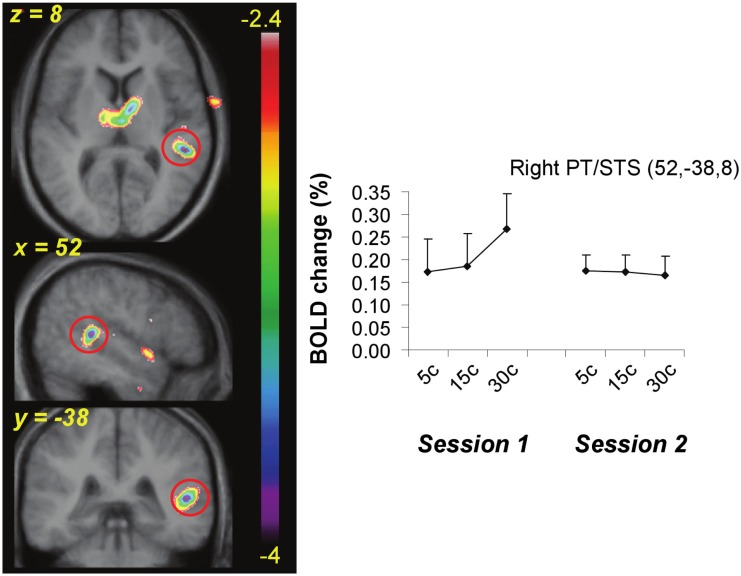
**Functional MRI data from the active task showing the site of maximal interaction between change in BOLD as a function of pitch interval size and effect of training**. A region near planum temporale and superior temporal sulcus (red circle) showed significant covariation with pitch interval size prior to training (Session 1) but a flat function after training (Session 2).

The equivalent contrast between post- and pre-training covariation data in the active condition yielded several changes meeting the FDR threshold; the largest was slightly posterior to the right planum temporale, near the superior temporal sulcus (52, −38, 8; *t* = 4.03), where there was maximum decrease in covariation with the input variable (Figure 6). The activity within this region was highly correlated with input at the pre-training session (in fact, there is a peak at the identical coordinate in the pre-training session, *t* = 4.7; Table 2) but there was no correlation with pitch interval size at the post-training session in this spot, hence yielding the significant interaction. This area was also one that showed one of the largest *R* > *L* asymmetries in the covariation analysis in the pre-training session. Two other auditory cortical areas also showed a negative change in this analysis, one in a roughly symmetrical position on the left side (−40, −42, 14, *t* = 2.58), and another in the right anterior STG (53, 4, −5; *t* = 2.85). To confirm the findings from the whole-brain analysis that there was a decrease in the relation between pitch interval size and activity after learning in the active task, we calculated the change in slope of the BOLD signal from before to after learning for two anterior STG sites, one on the left and one on the right, identified independently in the pre-training data on the basis that they yielded the largest covariation effect (Table 2); the results indicated that both areas showed a significant decrease in slope [*t*(9) = 1.82 and 1.95; *p* = 0.05 and 0.04 respectively].

#### Relation to speed of learning

**Figure 7:**
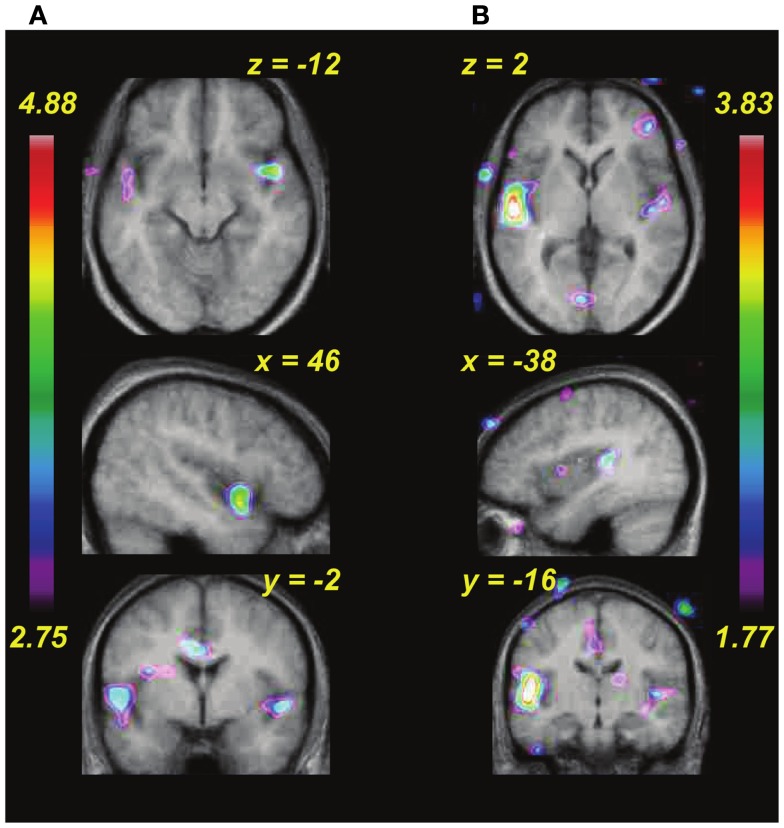
**Functional MRI data showing changes in covariation of BOLD activity with increasing pitch interval size during pre-training as a function of the individual rate of learning during training (i.e., behavioral data shown in Figure 4)**. **(A)** Passive task. **(B)** Active task. In both tasks, auditory cortical areas respond more to increasing pitch interval size in those individuals who subsequently showed faster learning.

The next step in the analysis was to examine the relation between brain activity patterns and behavioral learning performance. Given the differences in learning rate described above (Figure 4), we wondered whether anything in the brain activity patterns prior to the start of training would be predictive of subsequent learning speed. To evaluate this question on a whole-brain level, we entered each individual’s behavioral learning slope as a regressor in covariation analyses testing the relation between pitch interval size and BOLD response (as above). For both passive and active tasks in the pre-training scan session (Figures 7A,B), we found that there was a significantly greater degree of covariation in response to increasing pitch interval size as a function of behavioral learning slope in several auditory cortical areas bilaterally (for the passive task, in the anterior right STG: 46, 6, −12; *t* = 4.36, and in the anterior left STG: −52, −4, 0; *t* = 3.64; for the active task in the left Heschl’s gyrus: −52, −20, 8; *t* = 5.00, and a roughly symmetrical but non-significant effect in the right STG: 45, −16, 4; *t* = 2.43). Thus, those who subsequently proved to be faster learners, that is, those whose learning slope was relatively flat because they essentially reached maximal performance on the first day or two, generally showed a greater BOLD response to increasing pitch interval size, compared to slower learners, those who showed slow but steady improvement over the 6 days of training. There were no such effects in analyses examining the relationship between learning speed (learning slope entered as a covariate) and passive or active task versus silence, however, indicating that the individual differences were not in global level of activation, but rather in the degree to which the brain activity was a function of the input parameter, that is, pitch interval size.

**Figure 8:**
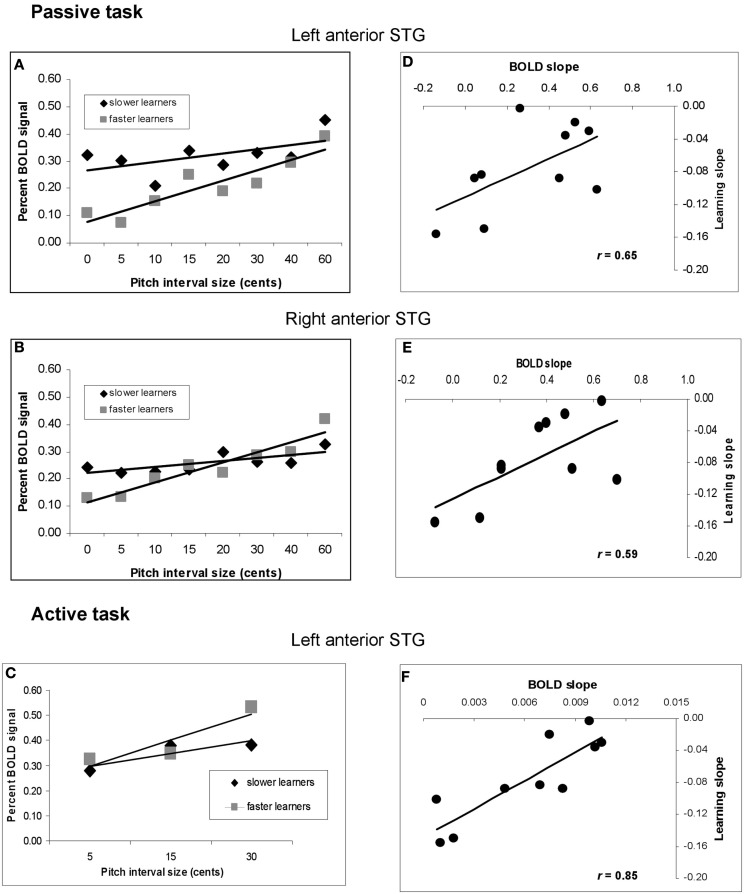
**Analysis of relationship between speed of learning during training and pre-training BOLD response to pitch interval size**. **(A)** Function relating BOLD signal to increasing pitch interval size in the passive task, divided according to the two subgroups of faster and slower learners. fMRI data extracted from left anterior STG site based on the pre-training covariation analysis (red circle in Figure 5A). **(B)** As in **(A)**, using fMRI data from the right anterior STG. **(C)** As in **(A)** using data from the active task; left anterior STG location. In all three cases, the slope is steeper in the faster learners. **(D)** Scatter plot showing the relationship between slope of BOLD response to increasing pitch interval size pre-training (abscissa) and behavioral learning slope (ordinate) at the same right anterior STG region as in **(A)**. Each symbol represents one individual. **(E)** As in **(D)**, but for the right anterior STG region corresponding to **(B)**. **(F)** As in **(D)** but for the active task, left anterior STG location corresponding to **(C)**. The significant correlations in all three cases indicate that the higher the individual BOLD response was to increasing pitch interval size prior to training, the faster the behavioral thresholds dropped to asymptotic levels during training.

To confirm and further understand these effects, we calculated the slope of the BOLD response as a function of increasing pitch interval size for the two subgroups (slower and faster learners). To avoid circularity, we extracted the BOLD signal from STG regions identified independently in the pre-training covariation analyses for both passive and active tasks (Figure 5), which are unbiased with respect to the two subgroups. This analysis (Figures 8A,B) demonstrated that for the passive task, the BOLD response to increasing pitch interval size prior to learning was steeper on average in the faster learners in a left anterior STG location (−56, −10, 4; *t* = 2.28, *p* < 0.04), with a similar trend in a symmetrical right STG location (58, −2, −2; *t* = 1.81, *p* < 0.07). Note that the effect is related to BOLD signal slope, and not to overall magnitude of BOLD response. To demonstrate more directly that the BOLD signal slope was truly related to individual speed of learning, we calculated a correlation between the learning slope for each person and the BOLD signal slope pre-training for the passive task at the same two anterior STG locations (Figures 8D,E). In both cases the correlation is significant (left: *r* = 0.65, *p* < 0.03; right: *r* = 0.59, *p* < 0.05) indicating that the steeper the BOLD response prior to learning, the faster the thresholds dropped during learning. Similar analyses were carried out for the active task, with similar results. The faster learners had a steeper BOLD slope to increasing pitch interval size (Figure 8C) at a left anterior STG site (−58, −6, 2; *t* = 2.51, *p* < 0.03) and a trend in this direction at a posterior right STG site (data not shown; 52, −38, 8; *t* = 1.59, *p* < 0.08). The slope of the BOLD response at the left STG site (Figure 8F) also predicted learning slope significantly (*r* = 0.85, *p* < 0.001).

#### Global changes associated with training

**Figure 9:**
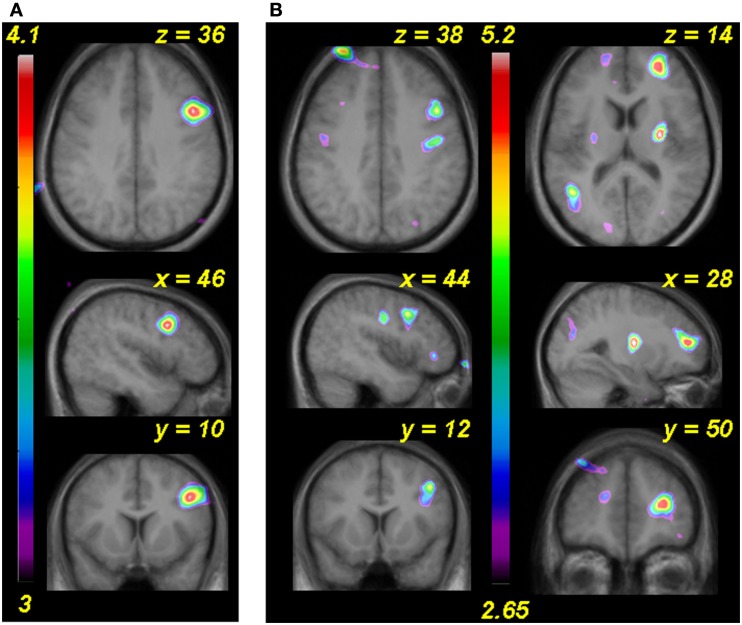
**(A)** Functional MRI data for the analysis comparing post-training to pre-training global BOLD changes in the passive task versus silence. **(B)** As in **(A)** but for active task. In both cases an area of the right dorsolateral frontal cortex shows more activity after training.

All of the above fMRI analyses were designed to probe the specific neural correlates of the pitch interval manipulation within the micromelodies, which was the principal hypothesis of interest. However, it is also relevant to ask what other, more global effects may have been associated with training. In order to examine these more non-specific effects, we performed simple contrasts between post- and pre-training for the two principal stimulation conditions, passive and active (as compared to silent baseline), collapsing across all pitch interval conditions. Thus, this analysis simply reveals brain regions whose BOLD signal either increased or decreased in a global manner following training. Looking first at the passive condition, we observed significant BOLD decreases bilaterally in several anterior STG regions (−50, −6, 4; *t* = 3.74 and 66, −2, 8; *t* = 3.45), reflecting the global changes observable in the graphs in Figure 5 (data not shown). In addition there was a single significant BOLD increase in the right dorsolateral frontal cortex (46, 10, 36; *t* = 5.16; Figure 9A). Similarly, in the active condition we observed significant signal decreases (data not shown) in right anterior STG (66, −6, 2; *t* = 4.59) and in the right Heschl’s gyrus (50, −20, 8; *t* = 3.42). There were simultaneous increases (Figure 9B) in the right anterior frontal (28, 50, 14; *t* = 4.08) and dorsolateral frontal (44, 12, 38; *t* = 3.71) cortex; although the latter did not reach the FDR level of significance, the fact that it lies within millimeters of its counterpart in the passive task suggests that it is unlikely to be a false-positive. Inspection of the separate contrasts before and after training revealed that the frontal changes were primarily due to relative deactivation in these regions prior to training, which reversed to a low level of activity post-training; in contrast, the signal in STG was strongly positive in both pre-training contrasts separately, and the decrease observed when comparing them is hence due to a lower level of overall recruitment following training.

## Discussion

To summarize the principal findings: we showed that the learning procedure was effective, and that behavioral improvement generalized to untrained frequencies and tasks (Figure 3). Increasing BOLD signal to increases in pitch interval size was seen in AC, as predicted (Figure 5). Following training, this effect diminished throughout the AC, with maximum reduction in right posterior AC (Figure 6). Thus, training specifically reduced the degree to which AC was modulated by the size of pitch intervals. As well, we found a predictive relationship between pre-training BOLD response to increasing pitch intervals and subsequent speed of learning, such that those individuals who had a relatively steeper AC response function to pitch variation prior to learning subsequently learned more rapidly than those with shallower functions (Figures 7 and 8). We also found two more global changes: overall STG response to all stimuli compared to silence diminished after training; and a region within the right dorsolateral frontal cortex was recruited to a greater extent after training in both tasks (Figure 9).

### Task validation

Several aspects of the results validate the experimental approach. First, looking just at the behavioral data, we found, as expected, a significant effect of training comparing performance from pre- to post-training time points (Figure 3). Moreover, the training generalized to other frequencies and to a related task. This generalization is in keeping with predictions based on prior behavioral studies (Demany, 1985; Delhommeau et al., 2002, 2005; Ari-Even Roth et al., 2003). Importantly, learning was not restricted to a specific frequency range, hence implicating configural learning mechanisms, as suggested by the cognitive literature on melody processing (Divenyi and Hirsh, 1978; Dowling, 1978). The lack of learning in the control sample confirms that the training procedure was effective, and that the changes in the experimental group were specific to training.

A potential concern with regard to the control sample is whether neural changes might have occurred despite the lack of training. Many studies have shown that the mere passage of time does not typically result in systematic changes in neural activity of the type seen in our trained sample (Wei et al., 2004; Aron et al., 2006; Gonzalez-Castillo and Talavage, 2011) so that possibility can likely be ruled out. Gaab et al. (2006) specifically included an untrained control group in their study of auditory working memory training and reported no change in AC activity, consistent with our claim that AC changes are associated with training. Interestingly, they did see an increase in a frontal region despite the absence of training, a finding which highlights the value of dissociating non-specific effects from those that are directly stimulus-driven, as we have endeavored to do here by measuring BOLD signal as a function of pitch interval size. We cannot rule out the possibility that passive exposure to the stimuli, rather than active training, might have resulted in changes similar to those we observed in the trained group. However, there is much evidence from the animal literature that cortical plasticity is strongest when stimuli are behaviorally relevant and if tasks are actively trained (Recanzone et al., 1993; Fritz et al., 2005; Ohl and Scheich, 2005). There is also relevant evidence from human music training studies in which one group was trained to play the piano while a control group listened to the sounds made by the first group and detected errors in performance; the active training group showed larger changes in magnetoencephalographic potentials than did the control group (Lappe et al., 2008) again pointing to the importance of active training in inducing cortical plasticity.

Our claim for training-induced specificity is further strengthened by the findings in a companion paper (Zarate et al., 2010) in which no BOLD signal changes were detected in the same subject sample tested here using a vocal micromelody production task. Importantly, no transfer of learning occurred from the micromelody perception task (the task used here) to the vocal task; thus a lack of learning was associated with a lack of neural changes. The close link between behavioral improvement and changes in neural responses in the present study, coupled with the lack of such a relation in the prior study, constitutes an essential element allowing us to interpret the current findings as being specific to training.

Finally, an additional aspect of the results that validate the methods used is that the fMRI data (both in passive and active conditions), showed the predicted pattern (Hyde et al., 2008) of increased BOLD with increasing pitch interval (Figure 5). These responses encompassed both anterior and posterior STG, but not peri-primary areas in Heschl’s gyrus, in accord with prior studies (Griffiths et al., 1998; Patterson et al., 2002). In addition, we observed a significantly higher slope in right compared to left AC, again as expected based on other studies in which pitch interval size was varied systematically (Zatorre and Belin, 2001; Jamison et al., 2006; Hyde et al., 2008). One interpretation of this asymmetry is that it reflects greater right AC resolution in the pitch domain (Zatorre et al., 2002), consistent with the microtonal stimuli used. Validation of behavioral and neural responses enhances interpretation of the effects associated with training.

### Effect of training

The principal neural change associated with training was a flattening of the function linking BOLD to pitch interval size, as well as an overall drop in activity (Figures 5 and 6). Decreased global activation has been observed in many learning studies (Kelly and Garavan, 2005), including one testing pitch learning (Jäncke et al., 2001); but interpretation of such effects is often difficult, particularly when behavioral changes are present, as is typically the case. In these circumstances it can be difficult to establish whether any changes are due to decreased attentional or other cognitive demands as the task becomes easier, to global familiarity with the stimuli, or to other non-specific effects (Poldrack, 2000). This problem is mitigated in the present study because rather than interpret the global decrease, which is subject to this ambiguity, we focus on the slope of the function that demonstrates sensitivity to pitch interval size. A change in slope means that there is a different response to some stimulus trials over others as a function of training. Non-specific effects, such as familiarity or changes in attention, would be expected to affect all stimuli equally; there is no reason to believe that attention would be systematically greater on some trials than on others; as for familiarity all stimuli would be equally familiar because they were all exposed to a similar extent during training. Therefore, the change in slope of the function cannot be attributed to these global factors, whereas the overall decrease in signal could be. Thus, we interpret the shallower slopes observed after training (seen in both conditions, but most clearly in the active condition) to be an indication that decreased neural activity is associated specifically with learning-related enhancement in processing the pattern of pitch-based information. In addition to these considerations, the fact that individual differences in the slope of the function was found to be predictive of subsequent learning rate is further evidence that this measure is specifically linked to the ability of interest, and not to some irrelevant factor.

The post-training change was stronger in the right hemisphere, consistent with related training data from electroencephalography measures (Shahin et al., 2003; Bosnyak et al., 2004), and was maximal near the right planum temporale, an area previously identified as important for pitch-pattern processing (Hyde et al., 2008). We propose that this training-induced modulation is related to the concept that certain regions of AC code for informational content in auditory patterns. Overath et al. (2007) reported less activity in right planum temporale when entropy in a tone sequence was low than when it was high. They concluded that planum temporale may be considered as “an efficient neural engine that demands less computational resource to encode redundant signals than those with high information content.” The relevance of this concept here is that following learning, the tonal patterns are presumably more efficiently encoded (leading to their enhanced discriminability) and therefore may be thought of not as containing less information *per se* – since this is a property of the stimulus itself – but rather as requiring less of the information to be processed to solve the task. Another, complementary way to consider the findings relates to the perceptual changes elicited by training. As noted by Werner (1940), training can result in a kind of perceptual expansion, such that intervals that are almost imperceptible prior to training become perceptually more comparable to larger intervals after training. This phenomenon could explain the changes in the slope of the BOLD signal: if smaller intervals are processed more like larger ones after training, then one would expect a flatter function since there is more equivalence across the different-sized intervals.

Having established a decreased cortical response to increasing pitch variation following training, we may ask how this phenomenon fits with neurophysiological observations of training-induced expansion in cortical representations (Recanzone et al., 1993; Polley et al., 2006). Although cortical expansion occurs for learning a single specific stimulus feature, this is not typically the case with patterned stimuli that cover a range of stimulus features, where reductions in cortical receptive fields have been observed without a change in the overall field map (Recanzone et al., 1992; Kilgard et al., 2001). Both enhancement and suppression of AC responses to melodies have been reported in trained monkeys (Yin et al., 2008). There is also evidence that perceptual learning is often accompanied by task-specific suppression of interfering neural signals (Ghose, 2004) and by decreased inter-neuronal noise correlations (Gu et al., 2011), which could result in lower overall activity. Given the many distinct neural response patterns reported, and the complex relationship between BOLD signal and electrophysiological responses (Logothetis and Wandell, 2004), it would be premature to attempt to link these phenomena directly to our present findings. Suffice it to say that with our task conditions a specific reduction exists in the relationship between BOLD response and the stimulus features that drive it. We interpret this to mean that fewer neuronal units are required to encode the same level of information, as also suggested for visual learning (Yotsumoto et al., 2008).

### Relation to individual differences in learning speed

This formulation of greater efficiency in the BOLD response after training may appear to be at odds with the additional novel finding that steeper slopes in the pitch-BOLD function prior to training were predictive of learning speed on an individual basis (Figures 7 and 8). If more efficient encoding entails a shallower slope, then why would steeper BOLD slopes to pitch change indicate greater propensity for learning rapidly? The answer we think is related to some aspect of cortical “state” or preparedness for learning, as distinguished from the effect of training itself. Our data indicate that the slope of the BOLD response pre-training has predictive validity with respect to behavioral learning speed, indicating that it is a relevant metric for perceptual processing. We suggest that the AC of faster learners initially encodes the relevant information with a greater degree of accuracy, or with higher resolution, which is why their BOLD signal tracks pitch changes more robustly. In turn, this cortical state endows them with greater learning readiness, such that a very short amount of exposure to the stimuli is sufficient to trigger enhanced behavioral discrimination, which then leads to the fast changes in threshold learning functions observed.

The source of these individual differences is unknown at the moment, but we note that two other studies of pitch learning have also identified subgroups who learn more or less well (Jäncke et al., 2001; Gaab et al., 2006) indicating that such individual differences can be observed under many circumstances. A number of neuroimaging studies have now begun to identify pre-existing anatomical features of auditory cortices that are predictive either of perceptual abilities (Schneider et al., 2002; Foster and Zatorre, 2010a), or of success and/or speed of learning (Golestani et al., 2002, 2007; Wong et al., 2008). Related findings have been reported in many other cognitive domains as well (Kanai and Rees, 2011). It seems likely that similar variation in brain anatomy or function – driven by experience, genetic, or epigenetic factors (or most likely by some combination) – could be related to the effects observed in the present study, and that is therefore a topic worthy of future investigation.

### Global increases

Post-learning decreases in the response function slope in auditory regions were accompanied by global increases in BOLD signal in right frontal regions in both passive and active tasks (Figure 9). The increased frontal activity might be related to tonal working memory, since similar regions are often recruited in tasks requiring listeners to encode and retain tones over short time periods (Zatorre et al., 1994; Holcomb et al., 1998; Gaab et al., 2003). This conclusion is in line with the well-established involvement of dorsolateral frontal cortex in aspects of working memory (Petrides, 2005) and is also consistent with frontal BOLD increases observed after training of working memory (Olesen et al., 2004). Interpretation of this finding must be approached cautiously, however, since as discussed above, various differences in how the task is performed could account for the change. Since there was no modulation of this area as a function of pitch size, however, we may safely infer that this region may participate in performance of the task, but is not directly related to perceptual learning.

## Conclusion

We conclude that pitch discrimination learning in the context of melodic patterns can be best understood in terms of more efficient encoding within AC regions sensitive to pitch patterns, such that fewer neural resources are required to process the same information as a consequence of learning. Responses within these areas prior to training are also predictive of learning potential. The dissociation between decreased response to increasing pitch interval size in AC, and globally increased response in the frontal cortex, points to the complex and dynamic nature of cortical changes associated with training, and also speaks to the interest of distinguishing non-specific effects from those specifically associated with the training-relevant stimulus features.

## Conflict of Interest Statement

The authors declare that the research was conducted in the absence of any commercial or financial relationships that could be construed as a potential conflict of interest.

## Supplementary Material

The Supplementary Material for this article can be found online at http://www.frontiersin.org/Auditory_Cognitive_Neuroscience/10.3389/fpsyg.2012.00544/abstract
